# The Pharma-Nutritional Role of Antioxidant Phytochemicals in Health and Disease

**DOI:** 10.3390/antiox11061081

**Published:** 2022-05-29

**Authors:** Sergio Davinelli, Giovanni Scapagnini

**Affiliations:** Department of Medicine and Health Sciences, University of Molise, 86100 Campobasso, Italy; giovanni.scapagnini@unimol.it

There is an increasing number of disease areas where nutritional and pharmacological applications complement each other [1]. Dietary phytochemicals comprise a large family of plant-derived molecules with highly diverse chemical structures, exerting biological activities in mammalian systems that may impact on health and disease risk [2]. These compounds are not directly involved in the growth, development, or reproduction of plants and they are synthesized to increase the survival fitness of the plant by allowing it to interact with its environment. For this reason, they are classified as secondary metabolites. Dietary phytochemicals are generally considered non-nutritive and many of the preventive effects of fruits and vegetables on chronic diseases have been attributed to specific phytochemicals found in these foods [3,4]. A growing body of evidence suggests that dietary phytochemicals can not only act as simple antioxidants agents that neutralize free radicals, but they can also modulate one or more cellular pathways associated with health and disease prevention. In fact, dietary phytochemicals or their gut-derived metabolites are known to interact with many biomolecules, especially proteins; hence, they may affect the function of enzymes, cell receptors or transcription factors. For example, the use of these bioactive molecules produces beneficial effects on physiological functions by activating several signalling pathways associated with mitochondrial function, inflammatory mediators, epigenetic changes, and expression of endogenous antioxidant enzymes [5,6,7]. Moreover, the large structural diversity makes dietary phytochemicals particularly attractive for drug discovery processes.

This Special Issue contains 15 articles covering several pharma-nutritional aspects of antioxidant phytochemicals.

There is some evidence that total dietary antioxidant capacity (TDAC) is inversely associated with the presence of obesity. However, no longitudinal studies have been performed investigating the effect of TDAC on comprehensive measures of body composition over time.

The results from the Rotterdam Study reveal that higher TDAC is associated with higher fat-free mass index in 4500 participants, suggesting that the increased consumption of antioxidants may have favorable effects on body composition and play a role in preserving lean mass over time [8].

Compared to other citrus fruits, bergamot has a higher concentration of polyphenols. This large family of antioxidant phytochemicals may modulate a variety of cellular processes, including mitochondrial function and sirtuin (SIRT) pathways. An animal study from Ilari et al. shows that restoring mitochondrial functions and protecting SIRT3 activity by bergamot polyphenols may be beneficial during oxidative stress-induced allodynia and hyperalgesia [9].

Raspberry ketones (RKs) are phenolic compounds found in red raspberries, kiwifruit, peaches, and apples. In vitro and in vivo studies have suggested an important role for RKs in hepatic/cardio/gastric protection and as an anti-hyperlipidemic and anti-obesity agent. The results from Mohamed at al. published in this Special Issue indicate that RKs attenuate cyclophosphamide-induced pulmonary toxicity in mice through the inhibition of oxidative stress and the nuclear factor kappa B (NF-κB) pathway [10].

Recently, scientific interest in dietary flavonoids has tremendously increased because of their postulated beneficial effects. Chrysin is a phytochemical categorized under the class of flavonoids and it is widely present in propolis, honey, passion fruit, and mushrooms. Using rats fed with high fructose corn syrup, Chang and colleagues showed that chrysin exerts its anti-hyperuricemic effect through antioxidant activity and inactivation of inflammasome, improving conditions of hyperuricemia-related metabolic disease [11]. Dietary flavonoids possess a large number of neuroprotective actions in various pathophysiological conditions. These compounds may promote synaptogenesis and neurogenesis, by, among other means, inhibiting oxidative stress and neuroinflammation. Cichon et al. have performed a comprehensive literature review describing the neurorestorative actions of flavonoids and how they can modulate brain plasticity processes. These authors discuss the latest research on the use of flavonoids as enhancers of neuroplasticity in the treatment of CNS diseases [12]. In this scenario, Ali et al. presents a systematic review and meta-analysis to evaluate whether flavonoids have some clinical efficacy in patients with depressive symptoms. They included 36 clinical trials involving a total of 2788 participants. The results indicate a significant antidepressant effect of flavonoids on the subjects affected by depressive symptoms [13].

Resveratrol is a non-flavonoid polyphenol that belongs to the stilbene family. Some clinical trials suggest that resveratrol may exert beneficial effects on metabolic diseases, which has been evidenced by its ability to reduce the levels of glucose. García-Martínez et al. conducted a systematic review and meta-analysis to analyze the hypoglycemic effect of resveratrol. The findings indicate that resveratrol improves glucose concentration, insulin levels, and glycated haemoglobin (HbA1c) in subjects with type 2 diabetes mellitus (T2DM) and aged 45–59 years [14]. However, despite these promising results, the low bioavailability of resveratrol reduces its efficacy and clinical translation. Resveratrol butyrate ester (RBE), which is a novel resveratrol-synthesized derivative, exhibits increased biological activity. Using a rat model of chronic kidney disease (CKD), Hsu et al. provides the first evidence that RBE may protect against kidney damage and hypertension by regulating the gut–kidney axis [15].

Among stilbenoid polyphenols, pterostilbene (PTS) stands out due to its high bioavailability. An experimental study in breast cancer cells by Harandi-Zadeh et al. delivers new knowledge about the effects of PTS on epigenetic marks at enhancer regions. They show an epigenetic silencing of oncogenes that may be an important contributor to the anti-cancer action of stilbenoid polyphenols [16].

Carotenoids belong to a large family of fat-soluble plant pigments that are widely present in yellow-orange vegetables and fruits. There is evidence that these compounds may positively affect cognitive function through their antioxidant and anti-inflammatory effects. A meta-analysis from our group explored the relationship between carotenoid supplementation and cognitive performance, using data from 9 intervention trials. Despite the small number of studies, the overall analysis indicates that carotenoid supplementation may help to improve cognitive performance in relatively healthy participants aged 45–78 years [17].

Seseli L. is one of the largest genera of the Apiaceae family, widely known for their traditional uses as herbal remedies. Zengin and colleagues conducted the first comprehensive biological and chemical evaluation of two seseli species (*S. gummiferum* and *S. transcaucasicum*). These two species are a rich source of polyphenolic compounds, especially chlorogenic acid and narcissin. The extracts display antioxidant effects, bactericidal activity, and act as inhibitors of enzymes involved in the pathogenesis of several diseases [18].

*Spirulina platensis*, a photosynthetic alga, is recognized as one of the most nutritious foods on the planet. It also contains high levels of antioxidants, including phycocyanin (PC). Omar et al. investigated effects of PC from *S. platensis* on growth, intestinal histomorphology (an indicator of gut health), blood biochemical parameters, antioxidant status, proinflammatory cytokines, and apoptotic proteins in broiler chicken. The study concludes that PC from *S. platensis* can be used as an alternative natural growth promoter, antioxidant, and anti-inflammatory feed additive for broilers production [19]

Nowadays, there is substantial evidence that phytoestrogens and their gut metabolites have the potential to prevent several chronic diseases. Phytoestrogens are polyphenolic estrogenic compounds of plant origin and are classified in the following five main classes: isoflavones, prenylflavonoids lignans, coumestans and stilbenes. Ionescu et al. provide an excellent overview of the new insights regarding the bioavailability and metabolism of dietary phytoestrogens. They also highlight emerging scientific data that sustain the capacity of each class of dietary phytoestrogens to modulate epigenetic processes [20].

*Withania somnifera* (Indian ginseng) comprises over 35 bioactive phytochemicals, including alkaloids, flavonoids, steroidal lactones, and saponins. However, since its discovery in the late 1960s, the oxygenated lactone withaferin A (WA) appears the most potent bioactive compound isolated from the roots of this plant. This compound has been identified as a promising anti-cancer and anti-inflammatory compound. Logie and Vanden Berghe provide an insightful overview, detailing how the polypharmacological mechanisms of action of WA may support the resolution of various chronic inflammatory diseases. They specifically emphasize how WA decreases the inflammatory response by interacting with the NF-κB pathway, protein kinases, heat shock proteins, and inflammasome activation [21].

Finally, Islam et al. present an updated review of the effects of dietary phytochemicals against human skin cancer. They describe how dietary phytochemicals may positively affect important cell signalling pathways involved in the progression of skin cancer. The authors also highlight gaps in the research, especially clinical validation [22].

Despite the relevance of the topics covered in the papers published in this Special Issue, many aspects remain relatively unexplored, including the complex relationship between dietary phytochemicals and gut microbiota.
